# Neural basis of visuospatial tests in behavioral variant frontotemporal dementia

**DOI:** 10.3389/fnagi.2022.963751

**Published:** 2022-08-23

**Authors:** Alfonso Delgado-Álvarez, María Nieves Cabrera-Martín, María Valles-Salgado, Cristina Delgado-Alonso, María José Gil, María Díez-Cirarda, Jorge Matías-Guiu, Jordi A. Matias-Guiu

**Affiliations:** ^1^Department of Neurology, Hospital Clinico San Carlos, San Carlos Institute for Health Research (IdiSSC), Universidad Complutense, Madrid, Spain; ^2^Department of Nuclear Medicine, Hospital Clinico San Carlos, San Carlos Institute for Health Research (IdiSSC), Universidad Complutense, Madrid, Spain

**Keywords:** frontotemporal dementia, visuospatial, Alzheimer’s disease, visual perception, positron–emission tomography

## Abstract

**Background:**

Recent models of visuospatial functioning suggest the existence of three main circuits emerging from the dorsal (“where”) route: parieto-prefrontal pathway, parieto-premotor, and parieto-medial temporal. Neural underpinnings of visuospatial task performance and the sparing of visuospatial functioning in bvFTD are unclear. We hypothesized different neural and cognitive mechanisms in visuospatial tasks performance in bvFTD and AD.

**Methods:**

Two hundred and sixteen participants were enrolled for this study: 72 patients with bvFTD dementia and 144 patients with AD. Visual Object and Space Perception Battery Position Discrimination and Number Location (VOSP-PD and VOSP-NL) and Rey-Osterrieth Complex Figure (ROCF) were administered to examine visuospatial functioning, together with a comprehensive neuropsychological battery. FDG-PET was acquired to evaluate brain metabolism. Voxel-based brain mapping analyses were conducted to evaluate the brain regions associated with visuospatial function in bvFTD and AD.

**Results:**

Patients with AD performed worst in visuospatial tasks in mild dementia, but not at prodromal stage. Attention and executive functioning tests showed higher correlations in bvFTD than AD with ROCF, but not VOSP subtests. Visuospatial performance in patients with bvFTD was associated with bilateral frontal regions, including the superior and medial frontal gyri, supplementary motor area, insula and middle cingulate gyrus.

**Conclusion:**

These findings support the role of prefrontal and premotor regions in visuospatial processing through the connection with the posterior parietal cortex and other posterior cortical regions. Visuospatial deficits should be interpreted with caution in patients with bvFTD, and should not be regarded as hallmarks of posterior cortical dysfunction.

## Introduction

Frontotemporal dementia is the second most common cause of dementia in adults under 65 years (Olney et al., 2017). The behavioral variant of frontotemporal dementia (bvFTD) is commonly associated with personality changes (Pennington et al., 2011) and cognitive symptoms, where social cognitive and executive dysfunction have been described as the hallmarks of the bvFTD (Laisney et al., 2009).

Alzheimer’s Disease (AD) is the most common cause of dementia, typically associated with a posterior pattern of neurodegeneration and prominent episodic memory impairment (Alzheimer’s Association, 2019). Differential diagnosis of bvFTD and AD may be challenging, especially considering the presence of executive functioning and episodic memory deficits in both cases (Fernandez-Matarrubia et al., 2017; Salimi et al., 2019). In this regard, the assessment of visuospatial skills has been proposed as a possible measure to distinguish AD from bvFTD, and relative sparing of visuospatial function is specifically recognized as one of the neuropsychological features of bvFTD in the international consensus criteria for this disorder (Rascovsky et al., 2011). Conversely, recent evidence has shown the limited ability of visuospatial tasks to differentiate between AD and bvFTD (Grossi et al., 2002; Salimi et al., 2019). However, visuospatial tasks could have a role in the differential diagnosis between bvFTD and AD when considering the clinical stage and the results of other neuropsychological tests (Ranasinghe et al., 2016; Salimi et al., 2019; García-Gutiérrez et al., 2021). While visuoconstruction deficits seem to appear at early stages of bvFTD, other visuospatial skills are relatively preserved and show a low performance with advancing disease. This highlights the importance of considering disease stages when interpreting patients’ performance on neuropsychological tests (Ranasinghe et al., 2016; Miyagawa et al., 2020).

Some studies have also reported differences in visuospatial tasks performance between FTD variants (Floris et al., 2015; Chen et al., 2020). For instance, progranulin mutation carriers showed worst performance in working memory and visuospatial functioning than non-carriers, and this mutation has been associated with parietal damage, often asymmetrical (Hallam et al., 2014). Whether executive function or an actual visuospatial dysfunction is causing the low performance in visuospatial tasks in bvFTD is under debate (Hallam et al., 2014; Floris et al., 2015).

Visuospatial functioning involves different brain regions and networks, from a basic level of perception more related to occipital cortices, to a more complex and extended level of integration associated with temporal, parietal, and frontal regions based on the ventral (also called “what”) and dorsal (also called “where”) paths (Lezak et al., 2012; Salimi et al., 2018). Recently, the role of ventrolateral prefrontal and dorsolateral prefrontal cortex has been emphasized in spatial working memory, visually guided actions, and navigation (Kravitz et al., 2011). To our knowledge, previous studies analyzing the neural correlates of visuospatial functioning in bvFTD have mainly based on the Rey-Osterrieth Complex Figure (ROCF) (Possin et al., 2011; Chen et al., 2020), but have not used more specific visuospatial tasks.

We hypothesized that neural and cognitive correlates of visuospatial tasks are different between bvFTD and AD. While in AD it could be a marker of posterior cortical dysfunction, in bvFTD they should be associated with frontal regions associated with visuospatial functioning. In addition, we hypothesized that the influence of inhibitory and executive control could have a relevant role in tasks used for visuospatial examination in bvFTD.

Our aim was to describe the neural correlates of visuospatial abilities in bvFTD patients and compare them with AD patients. To this end, we examined a large cohort of patients with bvFTD and AD that underwent cognitive assessment and FDG-PET imaging. Voxel-based brain mapping analysis was conducted to define the main regions associated with visuospatial skills in these disorders.

## Materials and methods

### Participants

Two hundred and sixteen participants were enrolled for this study: 72 patients with behavioral variant frontotemporal dementia and 144 patients with Alzheimer’s disease. Main clinical and demographic characteristics are depicted in Table 1.

**TABLE 1:** Demographic, main clinical characteristics, and visuospatial performance.

		bvFTD	AD	*t* or *X^2^, p*
*N*		72	144	−
Age, years		71.33 ± 7.75	73.32 ± 6.89	*t* = −1.913, *p* = 0.057
Female,%		40.3%	59.1%	*X*^2^ = 5.795, *p* = 0.016*
Education, years		9.22 ± 4.29	9.69 ± 4.80	*t* = −0.708, *p* = 0.482
Age of symptoms onset		66.57 ± 8.50	70.95 ± 6.46	*t* = −2.478, *p* = 0.016*
Global CDR	0.5	−	80 (55.6%)	−
	1	−	64 (44.4%)	
Global CDR plus NACC FTLD	0.5	16 (22.2%)	−	−
	1	28 (38.9%)	−	
	2	22 (30.6%)	−	
	3	6 (8.3%)	−	
MMSE, total score		23.77 ± 4.77	22.80 ± 4.83	*t* = 1.216, *p* = 0.226
ACE-III, total score		65.37 ± 16.65	67.06 ± 16.16	*t* = −0.073, *p* = 0.942
FAQ, total score		13.21 ± 9.86	10.69 ± 8.46	*t* = 1.216, *p* = 0.226
VOSP-PD		18.14 ± 2.61	18.37 ± 2.72	*t* = −0.577, *p* = 0.565
VOSP-NL		7.11 ± 3.10	7.24 ± 2.94	*t* = −0.297, *p* = 0.767
ROCF		21.42 ± 9.89	25.02 ± 18.53	*t* = −1.514, *p* = 0.132

*p < 0.05.

**FIGURE 1:**
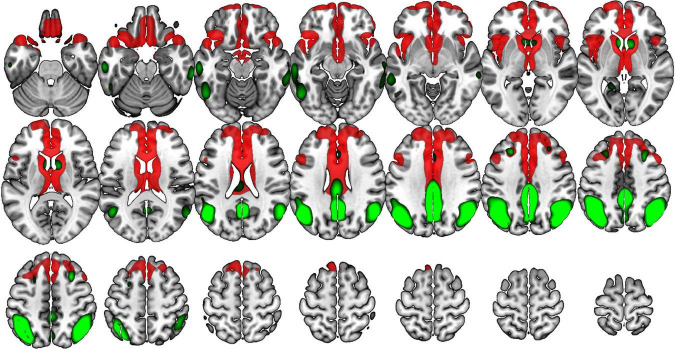
Voxel-based brain mapping analysis showing brain regions with lower metabolism in the group of bvFTD (red) and AD (green) compared with a healthy control group (FEW-peak corrected *p*-value < 0.05). Images are shown in neurological orientation.

Patients with bvFTD met current diagnostic criteria (Rascovsky et al., 2011) and the diagnosis was supported by FDG-PET findings and, at least, 2 years of follow-up. Patients with AD were diagnosed after a clinical and neuropsychological protocol, including confirmation by biomarkers (FDG-PET or CSF), and clinical progression during the follow-up (McKhann et al., 2011). Patients with any kind of motor dysfunction (e.g., parkinsonism, tremor, motor neuron disorder) were excluded. In all cases, the predominant symptom at presentation was memory loss. For descriptive purposes, we compared the brain metabolism of each group against 40 healthy controls (Figure 1).

Patients were evaluated with the Mini Mental State Examination (Folstein et al., 1975), the Addenbrooke’s Cognitive Examination III (Matias-Guiu et al., 2016), as measures of global cognition, and a comprehensive neuropsychological battery co-normed and validated in our setting (Peña-Casanova et al., 2009). This battery was composed of: forward and backward verbal span, forward and backward visual span (Corsi’s test), Trail Making Test (TMT), Symbol Digit Modalities Test (SDMT), Stroop Color-Word Interference Test, Boston Naming Test (BNT), semantic (animals) and phonemic (words beginning with “p”) fluency, Free and Cued Selective Reminding Test (FCSRT), and Tower of London-Drexel test (ToL). In addition, global CDR was used for staging of AD patients (Morris, 1993), and global CDR plus NACC FTDL rating for patients with bvFTD (Miyagawa et al., 2020). Functional Activities Questionnaire (FAQ) (Olazarán et al., 2005) was administered to evaluate daily living activities.

### Visuospatial assessment

The visuospatial assessment consisted of the Visual Object and Space Perception Battery: position discrimination (VOSP-PD) and number location (VOSP-NL) (Warrington and James, 1991) and the ROCF test (Rey, 1944).

VOSP-PD consists of 20 items, where participants are shown two white squares (always in horizontal position), each one with a black point. Participants are asked which one has the point exactly located in the middle of the square (right or left square). One point is assigned for correct answers with a maximal of 20 points. There was no time limit, and it was not allowed to touch the stimuli.

VOSP-NL consists of 10 items, where participants are shown two white squares (vertical position): the upper square shows different numbers in different positions, while the bottom square shows a black point in a position that coincides with the position of a number in the upper square. Participants are asked to say which number matches the point according to its position. There was no time limit, and it was not allowed to touch or move the stimuli.

ROCF test has four parts: copy task, free recall at 3 min, at 30 min, and a recognition task. For the visuospatial scores, only the copy task was considered. Participants were given a draw of the ROCF and a white page (always in horizontal position) to copy the figure. Figure accuracy, correct position, constructional strategy, and time in seconds were scored, according to the manual (Rey, 1997). The ROCF copy task has 18 items and a maximal score of 36 points, where each item has a maximal score of 2 points (1 considering the accuracy of the drawn item + 1 if the drawn item was placed in the correct position). Margins could not be part of the draw, second attempts were allowed (at the end, participants were asked to choose the best draw to be assessed), and there was no time limit. ROCF is considered a test for the examination of perceptual abilities and constructive praxis, although executive functioning is also involved (Lezak et al., 2012).

### ^18^F-FDG-PET acquisition, preprocessing, and analysis

Statistical Parametric Mapping version 12 (The Wellcome Trust Centre for Neuroimaging, Institute of Neurology, University College of London)^1^ was used for preprocessing and analysis of FDG-PET imaging. The images were manually realigned to the bicommissural line. Normalization to the reference space of the Montreal Neurological Institute was conducted using the validated FDG-PET template for dementia (Della Rosa et al., 2014). Then, images were smoothed using 12 mm of full width at half maximum. Cerebellum was used for intensity normalization (Dukart et al., 2013). A multiple regression analysis was conducted to evaluate the neural basis of each visuospatial task. In this analysis, age, sex, and years of education were entered as nuisance covariates. The analyses were conducted in the bvFTD and AD groups separately, and the statistically significant clusters were represented on an MRI template using the software MRIcroGL.^2^ A FWE-cluster corrected *p*-value < 0.05 was used for multiple comparisons correction.

### Standard protocol approvals and patient consents

All procedures performed were in accordance with the ethical standards of the institutional research committee and with the 1964 Helsinki declaration and its later amendments. The local Research Ethics Committee approved the research protocol.

### Statistical analysis

All statistical analyses were performed using SPSS Statistics 22.0. Descriptive data are shown as mean ± standard deviation. For intergroup differences, Student’s *t*-test was calculated. Eta squared was calculated for the measurement of effect size, considering the effect as small (eta squared = 0.010), moderate (0.058) and large (0.137). Chi-squared test was calculated for categorical variables. Correlation coefficients of Pearson and determination coefficients were calculated. Correlation coefficients were regarded as low (<0.30), moderate (0.30–0.49), or high (> 0.50). Fisher’s r-to-Z transformation was used to compare correlation coefficients. A *p*-value < 0.05 was considered statistically significant.

## Results

### Visuospatial performance

There were no statistically significant differences between groups in age (*t* = −1.913, *p* = 0.057), years of education (*t* = −0.708, *p* = 0.482) or functional impairment (FAQ: *t* = 1.448, *p* = 0.150). There were no statistically significant differences in VOSP-PD (*t* = −0.577, *p* = 0.565), VOSP-NL (*t* = −0.297, *p* = 0.767) and ROCF (*t* = −1.514, *p* = 0.132) between AD and bvFTD (Supplementary Table 1). When the analysis was segregated by CDR, there were no statistically differences in the 0.5 stage. In the CDR = 1 stage, patients with AD showed a lower scoring in VOSP-NL and ROCF (Table 2). For the other cognitive tests, at global CDR = 0.5, patients with AD scored lower on verbal and visual memory tests, and patients with bvFTD showed a shorter time to respond to ToL, suggesting impulsivity in the responses. At the stage of CDR = 1, there were significant differences in memory tests and also in the BNT, SDMT, and Stroop (especially trials 1 and 2) and TMT-A, with the worst performance in the AD group.

**TABLE 2:** Neuropsychological profile of bvFTD and AD.

Test	bvFTD (CDR 0.5)	AD (CDR 0.5)	bvFTD (CDR 1)	AD (CDR 1)	*t* (*p*)*	t (p)**
*Visuospatial tests*						
VOSP PD	19.31 ± 1.19	18.83 ± 2.05	18.86 ± 1.71	17.79 ± 3.30	0.90 (0.369)	1.70 (0.092)
VOSP NL	8.69 ± 1.58	8.06 ± 2.18	8.36 ± 1.61	6.23 ± 3.42	1.08 (0.283)	3.22 **(0.002)**
ROCF (copy accuracy)	25.96 ± 9.17	26.28 ± 7.23	24.59 ± 6.96	19.82 ± 8.10	−1.52 (0.880)	2.62 **(0.010)**
*Other cognitive tests*						
Verbal span—forward	5.44 ± 1.59	5.46 ± 1.07	5.37 ± 1.14	5.16 ± 1.28	−0.06 (0.953)	0.74 (0.457)
Verbal span—backward	3.81 ± 1.32	3.59 ± 0.96	3.37 ± 0.88	3.16 ± 1.14	0.79 (0.427)	0.86 (0.387)
Visual span—forward	4.63 ± 1.08	4.40 ± 0.94	4.29 ± 1.15	3.89 ± 0.98	0.85 (0.395)	1.68 (0.096)
Visual span—backward	4.06 ± 1.38	3.60 ± 1.06	3.21 ± 1.31	2.76 ± 1.10	1.49 (0.138)	1.70 (0.093)
TMT-A (seconds)	69.33 ± 25.37	83.05 ± 54.83	87.30 ± 47.26	130.87 ± 71.87	−0.94 (0.346)	−2.88 (**0.005)**
TMT-B (seconds)	183.63 ± 82.89	196.63 ± 80.92	236.78 ± 76.40	268.98 ± 58.94	−0.58 (0.561)	−1.95 (0.058)
SDMT	24.93 ± 10.87	23.05 ± 12.38	18.25 ± 12.22	10.00 ± 11.11	0.54 (0.585)	3.15 (**0.002**)
Stroop—reading	73.60 ± 21.59	79.48 ± 19.54	73.04 ± 22.36	57.00 ± 25.52	−1.04 (0.299)	2.66 (**0.009**)
Stroop—color naming	47.27 ± 8.53	48.19 ± 13.42	46.04 ± 15.89	35.57 ± 15.93	−0.25 (0.799)	0.87 (**0.010**)
Stroop—interference	23.20 ± 12.61	22.82 ± 10.26	21.00 ± 10.80	15.46 ± 10.48	0.12 (0.901)	2.11 (**0.038**)
ToL—correct score	2.94 ± 2.88	2.45 ± 2.08	1.70 ± 1.54	1.31 ± 1.67	0.79 (0.428)	1.05 (0.295)
ToL—total moves	52.86 ± 32.23	45.29 ± 27.49	58.11 ± 25.21	66.45 ± 52.85	0.90 (0.368)	−0.64 (0.522)
ToL—initiation time	51.21 ± 19.31	76.80 ± 52.70	60.85 ± 29.07	71.96 ± 34.42	−3.07 **(0.003)**	−1.17 (0.246)
ToL—execution time	412.79 ± 230.04	402.55 ± 178.37	489.50 ± 191.21	615.73 ± 258.96	0.18 (0.854)	−1.88 (0.065)
ToL—problem solving time	463.29 ± 228.32	480.44 ± 188.46	548.20 ± 196.16	669.73 ± 243.97	−0.29 (0.767)	−1.88 (0.065)
FCSRT—recall 1	4.25 ± 1.43	3.34 ± 2.39	3.48 ± 1.88	1.75 ± 1.46	1.46 (0.148)	4.70 **(<0.001)**
FCSRT—total free recall	16.13 ± 6.58	9.89 ± 7.22	9.59 ± 6.03	4.34 ± 4.21	3.19 **(0.002)**	4.11 **(<0.001)**
FCSRT—total recall	30.06 ± 9.68	22.27 ± 11.92	24.11 ± 11.49	10.42 ± 9.82	2.45 (**0.016)**	5.76 **(<0.001**)
FCSRT—delayed free recall	5.00 ± 3.18	2.47 ± 3.26	2.63 ± 2.96	0.66 ± 1.63	2.84 (**0.006**)	3.25 (**0.003)**
FCSRT—delayed total recall	9.69 ± 4.39	6.63 ± 4.66	7.07 ± 4.54	2.64 ± 3.60	2.51 (**0.020)**	4.50 **(<0.001)**
ROCF—3 min recall	12.23 ± 7.58	8.22 ± 6.03	7.32 ± 5.67	3.83 ± 3.20	2.24 (**0.027**)	2.92 (**0.006)**
ROCF—30 min recall	11.66 ± 6.97	7.18 ± 5.46	5.92 ± 4.60	2.43 ± 3.12	2.76 (**0.007**)	3.49 **(0.001)**
ROCF—recognition memory	16.69 ± 3.07	17.18 ± 3.07	15.72 ± 2.33	14.46 ± 3.81	−0.58 (0.558)	1.51 (0.133)
BNT	41.19 ± 11.34	42.95 ± 8.91	38.70 ± 9.38	30.48 ± 12.72	−0.68 (0.494)	3.01 (**0.003**)
Fluency (“*p*” words)	9.75 ± 5.20	12.33 ± 6.00	7.68 ± 4.72	7.08 ± 3.63	−1.59 (0.114)	0.66 (0.511)
Fluency (animals)	13.50 ± 5.42	14.61 ± 5.61	8.36 ± 4.08	9.68 ± 4.85	−0.727 (0.469)	−1.25 (0.212)

*Mean comparison between bvFTD (CDR plus NACC FTLD 0.5) and AD (CDR 0.5).

**Mean comparison between bvFTD (CDR plus NACC FTLD 1) and AD (CDR 1). Bold values are significant differences.

In bvFTD, scores on ROCF showed high correlations with VOSP-PD (*r* = 0.512, *p* < 0.001, *r*^2^ = 0.26) and VOSP-NL (*r* = 0.711, *p* < 0.001, *r*^2^ = 0.50). VOSP-PD was also highly correlated with VOSP-NL (*r* = 0.615, *p* < 0.001, *r*^2^ = 0.38). In AD, scores on ROCF showed a low correlation with VOSP-PD (*r* = 0.210, *p* = 0.016, *r*^2^ = 0.04). VOSP-NL was only significantly correlated with VOSP-PD (*r* = 0.494, *p* < 0.001, *r*^2^ = 0.24).

Patients with bvFTD showed high correlations between ROCF and semantic fluency, forward and backward digit span, forward Corsi test, TMT-A, TMT-B, Stroop (interference), and SDMT. Correlations with letter fluency, Corsi backwards, and Tower of London (total moves) were moderate. VOSP-PD showed moderate correlations with backward digit span, TMT-A, TMT-B, and SDMT. VOSP-NL showed high correlations with backward digit span, Corsi forward, TMT-A, TMT-B, SDMT, and moderate correlations with forward digit span, backward Corsi, Stroop (interference) and Tower of London (total moves).

**FIGURE 2:**
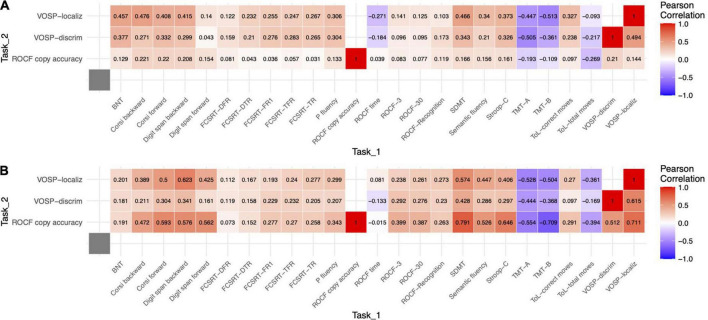
Heat map of Pearson correlations between visuospatial tasks and the other neuropsychological tests. **(A)** bvFTD. **(B)** AD.

Patients with AD showed a moderate correlation between ROCF and ToL. AD patients showed high correlations between VOSP-PS and TMT-A, and moderate correlations with forward Corsi test, TMT-B, SMDT, and Stroop (interference). Concerning VOSP-NL, it showed moderate correlations with backward digit span, forward and backward Corsi, TMT-A, SDMT, Stroop (interference), and Tower of London. Correlation with TMT-B was regarded as high. All correlations are depicted in Figure 2.

Correlation between ROCF and other tests was higher in bvFTD for VOSP-PD, VOSP-NL, digit span forward, digit span backwards, Corsi forward, TMT-A, TMT-B, SDMT, Stroop (interference), semantic fluency, and ROCF memory at 3 and 30 min. For VOSP-NL, the correlation was higher in digit span forward. Comparison of correlation coefficients of bvFTD and AD between VOSP-PD and other neuropsychological tests did not show any statistically significant result (Table 3 and Supplementary Table 2).

**TABLE 3:** Statistically significant comparisons of correlation coefficients of bvFTD and AD between visuospatial test and other neuropsychological tests.

Visuospatial test	Cognitive test	bvFTD	AD	Z	*p*
VOSP-PD	−	−	−	−	−
VOSP-NL	Span (F)	0.425 (0.005)	0.140 (0.101)	2.13	0.033
ROCF	Discrim	0.512 (<0.001)	0.210 (0.016)	2.4	0.016
	Localiz	0.711 (<0.001)	0.144 (0.101)	5.07	<0.001
	Semantic F	0.526 (<0.001)	0.156 (0.074)	2.91	0.003
	Span (F)	0.562 (<0.001)	0.154 (0.076)	3.27	0.001
	Span (B)	0.576 (<0.001)	0.208 (0.016)	3.03	0.002
	Corsi (F)	0.593 (<0.001)	0.220 (0.012)	3.12	0.002
	TMT-A	−0.554 (<0.001)	−0.193 (0.027)	−2.92	0.003
	TMT-B	−0.709 (<0.001)	−0.109 (0.212)	−5.28	<0.001
	SDMT	0.791 (<0.001)	0.166 (0.059)	6.17	<0.001
	ROCF-3	0.399 (0.001)	0.083 (0.52)	2.31	0.021
	ROCF-30	0.387 (0.001)	0.077 (0.389)	2.25	0.024
	Stroop C	0.646 (<0.001)	0.161 (0.074)	4.12	<0.001

Fisher’s r-to-Z transformation is shown to compare correlation coefficients.

### Voxel-based brain mapping analysis in behavioral variant of frontotemporal dementia and Alzheimer’s disease

**FIGURE 3:**
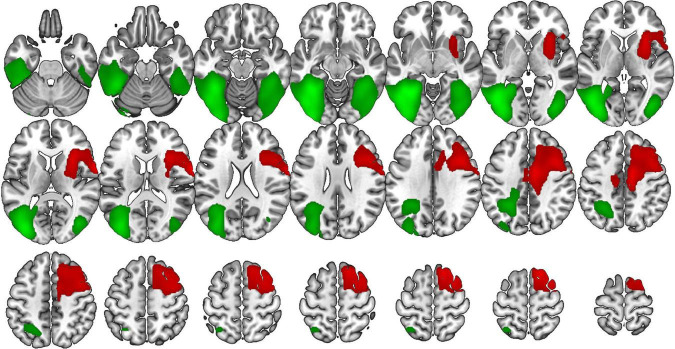
Voxel-based brain mapping analysis showing the correlation between the VOSP-PD and brain metabolism in bvFTD (red) and AD (green) (FWE-cluster corrected *p*-value < 0.05). Images are shown in neurological orientation.

In bvFTD, VOSP-PD was positively correlated with the right superior and inferior frontal gyri, medial frontal gyrus, supplementary motor area, insula, and middle cingulate gyrus. In AD, it was positively correlated with bilateral superior, middle, and inferior occipital gyri, fusiform and lingual gyri, middle temporal gyrus, and left parahippocampal gyrus (Figure 3).

**FIGURE 4:**
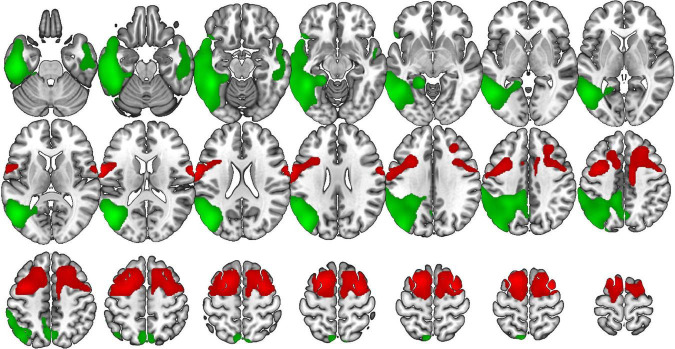
Voxel-based brain mapping analysis showing the correlation between the VOSP-NL and brain metabolism in bvFTD (red) and AD (green) (FWE-cluster corrected *p*-value < 0.05). Images are shown in neurological orientation.

VOSP-NL was positively correlated with the bilateral supplementary motor area, middle cingulate gyrus, precentral gyrus, medial frontal gyrus, and Roland operculum; and with the left superior, middle and inferior frontal gyri in bvFTD. While in AD, this test was positively correlated with the bilateral superior, middle, and inferior temporal gyri, bilateral precuneus, left superior and inferior parietal lobule, left lingual, fusiform, supramarginal, and angular gyri (Figure 4).

**FIGURE 5:**
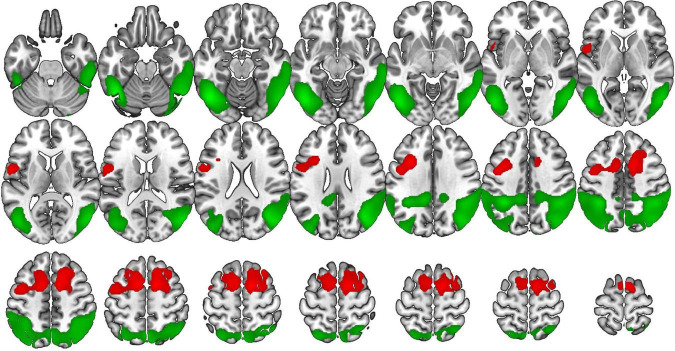
Voxel-based brain mapping analysis showing the correlation between ROCP (copy accuracy) and brain metabolism in bvFTD (red) and AD (green) (FWE-cluster corrected *p*-value < 0.05). Images are shown in neurological orientation (Left is left hemisphere, right is right hemisphere).

ROCF was positively correlated in bvFTD with the bilateral superior and middle frontal gyri middle cingulate gyrus, precentral gyrus, and supplementary motor area, and left superior temporal pole, inferior frontal gyrus, and insula. In AD, it was positively correlated with bilateral inferior and superior parietal lobule, inferior, middle and temporal gyri, precuneus, angular and supramarginal gyri, posterior cingulate and superior, middle, and inferior occipital gyri (Figure 5).

Complete details about statistics are shown in Supplementary Tables 3, 4.

## Discussion

The aim of our study was to describe the underpinnings of visuospatial performance in patients with bvFTD, compared with AD. The understanding of the cognitive processes and neural basis is key for an adequate interpretation of visuospatial tasks in the diagnosis of neurodegenerative disorders. Furthermore, the analysis of the neurocognitive and neural mechanisms associated with neuropsychological tasks is an excellent opportunity to evaluate the current models of brain function. Patients with bvFTD showed high and moderate correlations with several cognitive tests, especially with those more associated with attention and executive functioning. Specifically, correlations with attention and executive tests were higher in bvFTD than in AD for the ROCF, but not for the VOSP subtests. These findings are consistent with the current models about the processes involved in copying a complex figure such as the ROCF, which include the dorsal visual stream (analysis of localization of elements and spatial relations), ventral visual stream (recognition of stored elements, which facilitates the copy), prefrontal cortex (planning and monitoring of the copy), and premotor/motor regions (hand movement and dextral skills) (Trojano and Gainotti, 2016; Matías-Guiu et al., 2017). Importantly, the higher association with attention and executive functioning tests in bvFTD emphasizes that processes of planning and self-monitoring may have a more important role in the execution of ROCF in bvFTD than in AD. Thus, planning, strategy, monitoring, and absence of repetitions and perseverations during copy performance of each item are key in bvFTD. In contrast, there were no differences in the correlations between VOSP tasks and other tests between bvFTD and AD, which suggest that attention or executive function are not behind the deficits in these tests in patients with bvFTD. This means that scores in VOSP tasks are not influenced in a greater proportion by executive tests (at least the executive tests used in our study) in bvFTD than AD. However, correlations were statistically significant and generally moderate, suggesting the influence of executive function in visuospatial tasks in both disorders. In addition, we found that visuospatial task performance worsens in both AD and bvFTD when the dementia stage progresses, according to previous studies (Ranasinghe et al., 2016). Although we did not find statistically significant differences between bvFTD and AD groups in the visuospatial tests used in our study, the analysis of visuospatial performance in the context of the other tests and clinical staging could be helpful for differential diagnosis and monitoring (Ranasinghe et al., 2016; García-Gutiérrez et al., 2021). Impairment in visuospatial tests was greater in AD than bvFTD in the mild dementia stage but not in mild cognitive impairment stage, which suggests a different progression rate. To our knowledge, this study is the first to directly compare AD and bvFTD performance by disease stage, incorporating the global CDR plus NACC FTLD score for participants with a prominent behavioral syndrome consistent with the diagnosis of bvFTD. This finding indicates that considering disease stage is a critical point when interpreting the neuropsychological profile of patients with a prominent behavioral syndrome.

**FIGURE 6:**
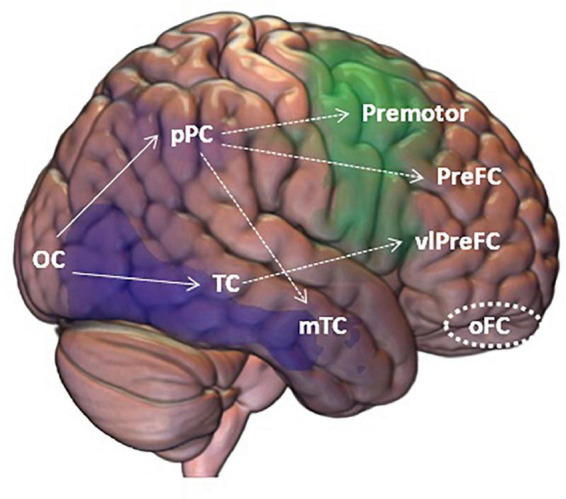
Framework for visuospatial processing and the regions and mechanisms producing visuospatial tasks impairment according to our findings in bvFTD (*green*) and AD (*violet*). OC, Occipital Cortex; Ppc, posterior Parietal Cortex; TC, Temporal Cortex; Premotor, Premotor Cortex; preFC, pre Frontal Cortex; mTC, middle Temporal Cortex; vlPreFC, ventrolateral prefrontal Cortex; oFC, orbitofrontal Cortex. Solid lines show the classic “what” and “where” pathways; dotted lines show the expanded pathways; circle shows inhibition areas that were not related to the scores on the visual tests.

The most remarkable findings of our study are the correlations with brain metabolism. In the bvFTD group, VOSP subtests were associated with the bilateral frontal lobe. Specifically, the superior and medial frontal gyri, supplementary motor area, insula and middle cingulate gyrus were involved. These findings are noteworthy because, for the first time to the best of our knowledge, they provide a rationale for the visuospatial findings encountered in patients with bvFTD. These results have important theoretical implications supporting the last models of visuospatial functioning based on the existence of three main circuits emerging from the dorsal visual stream (“where” route) (Figure 6): the parieto-prefrontal pathway, which is involved in the top-down control of eye movements and spatial working memory; the parieto-premotor pathway, which is associated with visually guided actions, the ability to maintain coordinated maps of the space and the 2D and 3D representations, including in the absence of visual guide (Schintu et al., 2014); and the parieto-medial temporal pathway, associated with spatial abilities related to navigation (Kravitz et al., 2011). Accordingly, in patients with bvFTD, low performance in visuospatial tasks and, specifically in VOSP subtests, could be explained by deficits in visually guided actions, spatial working memory and self-monitoring. These findings also have clinical consequences in the interpretation of neuropsychological assessments in bvFTD. According to our results, low scores in visuospatial tests should be interpreted with caution, and they should not be equated as posterior cortical dysfunction. This is especially important because, depending on the context, visuospatial deficits could be misinterpreted as a marker of posterior neurodegeneration due to Alzheimer’s disease neuropathological changes, corticobasal degeneration, or Lewy body disease (Bak et al., 2006).

Another important aspect is the influence of inhibitory control. Inhibition is the capacity to reject an automatic response. Inhibition has been mainly linked to the frontal cortex. In this regard, these regions may exert some sort of top-down control on the visual perception systems. Furthermore, disinhibition behavior could influence the task. The Hayling test and the Stroop test are among the most used tests to evaluate inhibition (Pérez-Pérez et al., 2016). Although we did not administer the Hayling test in this study, regions more associated with this test (i.e., the orbitofrontal cortex) (Matias-Guiu et al., 2019) are not associated with the visuospatial tasks. Regarding the Stroop, the correlation with VOSP subtests is not greater in bvFTD than AD. Thus, these findings suggest that inhibitory alterations are not playing an important role in the performance of the visuospatial tasks in bvFTD, rejecting one of our hypotheses.

In AD, VOSP subtests were mainly correlated with several regions in the parietal, temporal and occipital lobes. Both VOSP subtests used in our study should have been more associated with the dorsal pathway. However, brain regions associated from the voxel-based analysis are included within the dorsal and ventral pathways. This lack of specificity for the specific visual pathway has also recently been observed in a small cohort of 16 patients with non-amnestic forms of AD, in which VOSP-NL was correlated with tau deposition and atrophy in bilateral visual association areas, right hemisphere superior parietal cortex, and predominantly right hemisphere lateral and medial occipitotemporal cortex (Putcha et al., 2019). Overall, the findings in AD suggest the implication of the two main pathways of visual perception: the ventral (which extends from the ventral visual association cortex to the temporal cortex and drives visual perception) and the dorsal pathway (which extends from the dorsal visual association cortex to the posterior parietal cortex and drives visual attention and location). Recent studies have suggested that occipital correlation with visuospatial abilities could be suggestive of dementia with Lewy bodies, because the occipital lobe was correlated in dementia with Lewy bodies but not in AD (Beretta et al., 2019, 2022). However, these findings were observed with visuoconstructive tasks, according to our findings with ROCF. Future studies correlating VOSP with dementia with Lewy bodies could be of interest to evaluate potential differences in visuospatial processing compared with AD.

In bvFTD, metabolic correlates of ROCF overlap with the brain regions associated with VOSP. In this regard, correlation between VOSP and ROCF scores were high in bvFTD, suggesting common underlying mechanisms. In contrast, correlation between VOSP and ROCF was lower in AD, indicating more heterogeneous mechanisms involved in the execution of ROCF in AD than in bvFTD. Previous works examining the neural correlates of ROCF performance in AD have shown consistent results about the involvement of the right superior parietal lobe, and heterogeneous findings regarding the implications of bilateral temporal and frontal lobes and a potential predominance of the right hemisphere (Tippet and Black, 2008; Salmon and Bondi, 2009; Melrose et al., 2013). In our study, regions associated with ROCF in AD were mainly located in the bilateral parietal lobe, extending to the temporal and occipital lobes. The parietal lobe is well-known for key roles in spatial attention, orientation, and visuoconstructive praxis, and our study confirms this role in patients with AD. In contrast, in bvFTD, mechanisms associated with attention and executive functioning (inhibition capacity, cognitive flexibility, planning) and the frontal lobe have a more predominant role. In this regard, the analysis of qualitative findings (e.g., perseverations, figure rotations, etc.) could be of interest in future works to evaluate the regional and/or disease specificity of certain observations during the ROCF execution (Melrose et al., 2013).

Another interesting finding concerns hemispheric lateralization. Right hemispheric dominance seems to be prominent for visuospatial attention and perception, while dominance for object perception and recognition is still controversial (Bartolomeo and Malkinson, 2019). In our study, almost all tests showed a bilateral distribution, except VOSP-PD in bvFTD and VOSP-NL in AD. VOSP-PD correlated with the right hemisphere in patients with bvFTD, which is consistent with a study on traumatic brain injury, in which this test was linked to the right precentral gyrus, premotor area, precentral gyrus and insula using voxel-based lesion-symptom mapping (Schintu et al., 2014). As we included patients with bvFTD and amnestic AD, asymmetry in neuroimaging and focal onset is less frequent than in some variants of these disorders (e.g., posterior cortical atrophy, primary progressive aphasia). Thus, patients with focal damage could be more appropriate to evaluate the effects of hemispheric dominance for specific cognitive functions.

Our study has some limitations. First, we have focused on a quantitative analysis of the neuropsychological tests. In the case of the ROCF, a qualitative analysis could provide interesting information about patients’ performance. Second, we used only FDG-PET imaging. Although FDG-PET is a reliable approach to brain functioning, multimodal assessments combining PET and MRI may provide a more general view of brain regions and networks involved in a specific function (Matias-Guiu et al., 2021). Third, diagnoses of bvFTD and AD had no pathological confirmation, but pathophysiological biomarkers supported the diagnoses.

## Conclusion

In conclusion, our findings confirm the hypothesis that neural mechanisms underlying poor performance in visuospatial tasks is different in AD and bvFTD. Visuospatial performance in patients with bvFTD is associated with bilateral frontal regions, including the superior and medial frontal gyri, supplementary motor area, insula and middle cingulate gyrus. Influence of attention and executive functioning was greater in bvFTD than AD for the visuoconstructive task, but not for the visuospatial tests. These findings provide compelling evidence supporting the role of prefrontal and premotor regions in visuospatial processing through the connection with the posterior parietal cortex and other posterior cortical regions. Thus, visuospatial deficits should be interpreted with caution in patients with bvFTD, and should not be regarded as hallmarks of posterior cortical dysfunction.

## Data availability statement

The raw data supporting the conclusions of this article will be made available by the authors, without undue reservation.

## Ethics statement

The studies involving human participants were reviewed and approved by Comité de Ética e Investigación Clínica del Hospital Clinico San Carlos. Written informed consent to participate in this study was provided by the participants’ legal guardian/next of kin.

## Author contributions

MC-M, JM-G, and JAM-G: conceptualization and design of the study. AD-Á, MC-M, MV-S, CD-A, MD-C, MG, and JAM-G: data curation. AD-Á and JAM-G: formal analysis and writing—original draft. JM-G and JAM-G: funding acquisition and supervision. AD-Á, MC-M, and JAM-G: methodology. MC-M: writing—review and editing. All authors contributed to investigation.

## Footnotes

1
https://www.fil.ion.ucl.ac.uk/spm/software/spm12/

2
https://www.nitrc.org/projects/mricrogl/
