# Supplementary material for: Neural basis of visuospatial tests in behavioral variant frontotemporal dementia
Source: Front Aging Neurosci. 2022 Aug 23;14:963751. doi: 10.3389/fnagi.2022.963751 (PMC9445442; doi:10.3389/fnagi.2022.963751)
Supplement: Supplementary file 1 [file Table_1.docx]

Supplementary Table 1. *Neuropsychological profile of bvFTD and AD according to the Neuronorma battery.*

| Test | bvFTD | AD | *t* (*p*) |
| --- | --- | --- | --- |
| Verbal span – forward | 5.08 ±1.27 | 5.33 ±1.18 | -1.38 (.170) |
| Verbal span – backward | 3.06 ±1.26 | 3.40 ±1.07 | -2.06 (.040) |
| Visual span – forward | 4.06 ±1.17 | 4.17 ±0.99 | -0.75 (.455) |
| Visual span – backward | 3.17 ±1.41 | 3.23 ±1.15 | -0.33 (.739) |
| TMT-A (seconds) | 106.45 ±69.28 | 104.42±67.16 | 0.20 (.840) |
| TMT-B (seconds) | 237.51 ± 77.56 | 228.96 ±80.27 | 0.73 (.466) |
| SDMT | 16.72 ±12.54 | 17.28 ±13.47 | -0.29 (.774) |
| Stroop – reading | 63.00 ±26.55 | 69.87 ±24.85 | -1.78 (.076) |
| Stroop – color naming | 37.95 ±16.86 | 42.75 ±15.80 | -1.94 (.054) |
| Stroop – interference | 18.25 ±11.06 | 19.65 ±10.95 | -0.84 (.404) |
| ToL – correct score | 1.87 ±2.32 | 1.94 ±1.99 | -0.24 (.813) |
| ToL – move score | 63.07 ±42.57 | 52.42 ±39.01 | 1.48 (.140) |
| ToL – initiation time | 58.00 ±23.85 | 75.34 ±47.80 | -2.30 (.023) |
| ToL – execution time | 498.02 ±256.70 | 475.07 ±231.39 | 0.54 (.591) |
| ToL – problem solving time | 561.13 ±247.01 | 544.84 ±226.45 | 0.39 (.695) |
| FCSRT – recall 1 | 3.16 ±1.87 | 2.63 ±2.18 | 1.73 (.084) |
| FCSRT – total free recall | 9.68 ±7.13 | 7.41 ±6.65 | 2.26 (.025) |
| FCSRT – total recall | 22.49 ±12.50 | 16.97 ±12.48 | 3.00 (.003) |
| FCSRT – delayed free recall | 2.84 ±3.01 | 1.66 ±2.80 | 2.79 (.006) |
| FCSRT – delayed total recall | 7.07 ±4.94 | 4.85 ±4.66 | 3.18 (.002) |
| ROCF – 3 min recall | 7.45 ±6.22 | 6.36 ±5.47 | 1.26 (.208) |
| ROCF – 30 min recall | 6.76 ±5.61 | 5.17 ±5.17 | 1.99 (.048) |
| ROCF – recognition memory | 15.19 ±2.83 | 16.05 ±3.65 | -1.70 (.090) |
| BNT | 37.12 ±10.06 | 37.47 ±12.38 | -0.20 (.835) |
| Fluency ("p" words) | 6.83 ±4.73 | 10.01 ±5.72 | -4.07 (<.001) |
| Fluency (animals) | 8.74 ±4.80 | 12.44 ±5.82 | -4.66 (<.001) |
