# Supplementary material for: Neural basis of visuospatial tests in behavioral variant frontotemporal dementia
Source: Front Aging Neurosci. 2022 Aug 23;14:963751. doi: 10.3389/fnagi.2022.963751 (PMC9445442; doi:10.3389/fnagi.2022.963751)
Supplement: Supplementary file 2 [file Table_2.docx]

**Supplementary Table 2.** *Non-statistically* *significant* *comparisons of correlation coefficients of bvFTD and AD between VOSP-DP and other neuropsychological tests.* *Fisher's r-to-Z transformation is shown to compare correlation coefficients.*

|  |  |  |  |  |  |
| --- | --- | --- | --- | --- | --- |
| Visuospatial Test | Cognitive test | bvFTD | AD | Z | *p* |
| VOSP-PD | Span (B) | .341 (.004) | .299 (<.001) | 0.32 | .749 |
|  | Corsi (F) | .304 (.010) | .331 (<.001) | -0.2 | .841 |
|  | Corsi (B) | .211 (.078) | .271 (<.001) | -0.43 | .667 |
|  | TMT-A | -.444 (<.001) | -.505 (<.001) | 0.54 | .589 |
|  | TMT-B | -.368 (.002) | -.361 (<.001) | -0.05 | .960 |
|  | SDMT | .428 (<.001) | .343 (<.001) | 0.68 | .496 |
|  | FCSRT-TR | .205 (.094) | .267 (.002) | -0.45 | .652 |
|  | Stroop C | .297 (.017) | .326 (<.001) | -0.22 | .826 |
|  | ToL-CM | .238 (.005) | .238 (.005) | 0 | 1 |
| VOSP-NL | Span (B) | .623 (<.001) | .415 (<.001) | 1.96 | .05 |
|  | Corsi (F) | .500 (<.001) | .408 (<.001) | 0.79 | .429 |
|  | Corsi (B) | .389 (.001) | .476 (<.001) | -0.73 | .465 |
|  | TMT-A | -.528 (<.001) | -.447 (<.001) | -0.72 | .471 |
|  | TMT-B | -.504 (<.001) | -.513 (<.001) | 0.08 | .936 |
|  | SDMT | .574 (<.001) | .466 (<.001) | 1.01 | .312 |
|  | Stroop C | .406 (.001) | .373 (<.001) | 0.26 | .794 |
|  | FCSRT-TR | .277 (.023) | .267 (.002) | -0.73 | .465 |
|  | FCSRT-TDR | .167 (.176) | .232 (.006) | -0.46 | .645 |
|  | ToL-TM | -.361 (<.001) | -.093 (.364) | -1.94 | .052 |
|  | ToL-CM | .270 (.026) | .327 (<.001) | -0.43 | .667 |
| ROCF | Phonemic F | .343 (.004) | .133 (.129) | 1.52 | .128 |
|  | Corsi (B) | .472 (<.001) | .221 (.011) | 1.96 | .05 |
|  | ToL-TM | -.394 (.007) | -.269 (.008) | -0.96 | .337 |
