# Supplementary material for: Neural basis of visuospatial tests in behavioral variant frontotemporal dementia
Source: Front Aging Neurosci. 2022 Aug 23;14:963751. doi: 10.3389/fnagi.2022.963751 (PMC9445442; doi:10.3389/fnagi.2022.963751)
Supplement: Supplementary file 3 [file Table_3.doc]

| **Supplementary Table 3.** Voxel-based brain mapping analysis results in bvFTD.  Correlation with neuropsychological tests, using an uncorrected p-value <0.001 and a cluster size threshold k=50.  (*) means “negative correlation”; in other tests, positive correlations are shown. | | | | | | |  |
| --- | --- | --- | --- | --- | --- | --- | --- |
| Brain regions | MNI coordinates | | | T value | Z score | K (number of voxels) | r |
|  | x | y | z |  |  |  |  |
| *Regions correlated with* ***Rey-Osterrieth Complex Figure Test (Copy)*** | | | | | | |  |
| Right superior and middle frontal gyri.  Bilateral precentral and middle cingulate gyri, and supplementary motor area.  Left middle and superior frontal; opercular inferior frontal; postcentral; triangular inferior frontal gyri; Rolandic operculum; superior temporal pole; and insula. | 14 | 4 | 56 | 4.27 | 3.99 | 6975 | 0.45 |
|  | -32 | 12 | 32 | 4.16 | 3.90 |  | 0.44 |
|  | -12 | 6 | 52 | 4.12 | 3.87 |  | 0.44 |
| *Regions correlated with* ***Visual Object and Space Perception Battery (Position discrimination)*** | | | | | | |  |
| Right superior frontal; precentral; triangular inferior frontal; opercular inferior frontal; medial superior frontal gyri; putamen; supplementary motor area; insula; Rolandic operculum; and pallidum.  Bilateral middle cingulate gyrus. | 66 | 0 | 14 | 5.31 | 4.82 | 12919 | 0.53 |
|  | 12 | -6 | 42 | 5.13 | 4.69 |  | 0.52 |
|  | 16 | 20 | 64 | 4.76 | 4.40 |  | 0.49 |
| *Regions correlated with* ***Visual Object and Space Perception Battery (number location)*** | | | | | | |  |
| Right superior and middle frontal; precentral; opercular inferior frontal gyri.  Left precentral; middle and superior frontal; opercular inferior frontal; middle cingulate; triangular inferior frontal gyri; and paracentral lobe.  Bilateral supplementary motor area; postcentral; middle cingulate; medial frontal gyri; and roland operculum. | 32 | 2 | 64 | 4.93 | 4.52 | 12463 | 5.07 |
|  | -16 | 12 | 64 | 4.88 | 4.48 |  | 0.50 |
|  | -64 | 0 | 20 | 4.87 | 4.48 |  | 0.50 |
