# Supplementary material for: Neural basis of visuospatial tests in behavioral variant frontotemporal dementia
Source: Front Aging Neurosci. 2022 Aug 23;14:963751. doi: 10.3389/fnagi.2022.963751 (PMC9445442; doi:10.3389/fnagi.2022.963751)
Supplement: Supplementary file 4 [file Table_4.doc]

| **Supplementary Table 4.** Voxel-based brain mapping analysis results in AD.  Correlation with neuropsychological tests, using an uncorrected p-value <0.001 and a FWE-cluster based corrected p-value <0.05  (*) means “negative correlation”; in other tests, positive correlations are shown. | | | | | | |  |
| --- | --- | --- | --- | --- | --- | --- | --- |
| Brain regions | MNI coordinates | | | T value | Z score | K (number of voxels) | r |
|  | x | y | z |  |  |  |  |
| *Regions correlated with* ***Rey-Osterrieth Complex Figure Test (Copy)*** | | | | | | |  |
| Right inferior, middle and superior temporal, inferior, middle and superior occipital, inferior and superior parietal, precuneus, angular and supramarginal gyri, fusiform gyrus, middle cingulum gyri.  Left inferior and superior parietal, inferior and middle temporal gyri, inferior occipital, angular and supramarginal gyri, fusiform gyrus, precuneus, middle and posterior cingulate, supramarginal, lingual gyri. | **-44** | **-64** | **-18** | **5.99** | **5.61** | 31455 | 0.44 |
|  | **40** | **-68** | **24** | **5.33** | **5.05** |  | 0.40 |
|  | **-36** | **-46** | **40** | **5.28** | **5.01** |  | 0.40 |
| *Regions correlated with* ***Visual Object and Space Perception Battery***  ***(Discrimination of position)*** | | | | | | |  |
| Left inferior, middle and superior occipital, superior, middle and inferior temporal gyri, inferior and superior parietal, fusiform, lingual, parahippocampal gyri, precuneus. | **-34** | **-58** | **-8** | **6.49** | **6.04** | 15531 | 0.47 |
|  | **-44** | **-64** | **-2** | **6.34** | **5.91** |  | 0.46 |
|  | **-38** | **-82** | **8** | **6.05** | **5.67** |  | 0.45 |
| Right fusiform, inferior and middle temporal, inferior and middle occipital, parahippocampal gyrus, and lingual gyri. | **38** | **-78** | **0** | **5.05** | **4.82** | 6128 | 0.39 |
|  | **38** | **-52** | **-10** | **4.95** | **4.73** |  | 0.38 |
|  | 58 | -58 | -22 | 3.95 | 3.84 |  | 0.31 |
| *Regions correlated with* ***Visual Object and Space Perception Battery (number location)*** | | | | | | |  |
| Left inferior, middle and superior temporal, inferior and middle occipital, inferior and superior parietal lobule, angular, fusiform, precuneus, calcarine, supramarginal, middle cingulum, lingual, parahippocampal gyri, and middle and superior temporal pole.  Right precuneus. | **-44** | **-44** | **-18** | **5.62** | **5.31** | 22658 | 0.42 |
|  | **-46** | **-66** | **16** | **5.58** | **5.28** |  | 0.42 |
|  | **-48** | **-20** | **-30** | **5.50** | **5.21** |  | 0.41 |
| Right inferior and middle temporal gyri, and middle and superior temporal pole, fusiform gyrus. | 52 | 6 | -22 | 3.62 | 3.53 | 1674 | 0.29 |
|  | 50 | -32 | -18 | 3.60 | 3.51 |  | 0.28 |
|  | 48 | -16 | -30 | 3.47 | 3.39 |  | 0.27 |
